# PET Analysis of Dopaminergic Neurodegeneration in Relation to Immobility in the MPTP-Treated Common Marmoset, a Model for Parkinson’s Disease

**DOI:** 10.1371/journal.pone.0046371

**Published:** 2012-10-08

**Authors:** Kiyoshi Ando, Shigeru Obayashi, Yuji Nagai, Arata Oh-Nishi, Takafumi Minamimoto, Makoto Higuchi, Takashi Inoue, Toshio Itoh, Tetsuya Suhara

**Affiliations:** 1 Central Institute for Experimental Animals, Kawasakiku, Kawasaki, Kanagawa, Japan; 2 Rehabilitation Center, Nippon Medical School, Chiba Hokusoh Hospital, Insei, Chiba, Japan; 3 Molecular Imaging Center, The National Institute of Radiological Sciences, Inageku, Chiba, Japan; St. Jude Children’s Research Hospital, United States of America

## Abstract

**Background:**

Positron Emission Tomography (PET) measurement was applied to the brain of the common marmoset, a small primate species, treated with 1-methyl-4-phenyl-1,2,3,6-tetrahydropyridine (MPTP). The marmoset shows prominent Parkinson’s disease (PD) signs due to dopaminergic neural degeneration. Recently, the transgenic marmoset (TG) carrying human PD genes is developing. For phenotypic evaluations of TG, non-invasive PET measurement is considered to be substantially significant. As a reference control for TG, the brain of the MPTP-marmoset as an established and valid model was scanned by PET. Behavioral analysis was also performed by recording locomotion of the MPTP-marmoset, as an objective measure of PD signs.

**Methodology/Principal Findings:**

Marmosets received several MPTP regimens (single MPTP regimen: 2 mg/kg, s.c., per day for 3 consecutive days) were used for PET measurement and behavioral observation. To measure immobility as a central PD sign, locomotion of marmosets in their individual living cages were recorded daily by infrared sensors. Daily locomotion counts decreased drastically after MPTP regimens and remained diminished for several months or more. PET scan of the brain, using [^11^C]PE2I as a ligand of the dopamine (DA) transporter, was performed once several months after the last MPTP regimen. The mean binding potential (BP_ND_) in the striatum (putamen and caudate) of the MPTP-marmoset group was significantly lower than that of the MPTP-free control group (n = 5 for each group). In the MPTP-marmosets, the decrease of BP_ND_ in the striatum closely correlated with the decrease in locomotion counts (r = 0.98 in putamen and 0.91 in caudate).

**Conclusion/Significance:**

The present characterization of neural degeneration using non-invasive PET imaging and of behavioral manifestation in the MPTP marmoset mimics typical PD characteristics and can be useful in evaluating the phenotype of TG marmosets being developed.

## Introduction

Parkinson’s disease (PD) is a movement disorder caused by degeneration of nigrostriatal dopamine (DA) neurons in the brain and is thought to have several contributing factors, including genetic predisposition and environmental toxins [1]. Recent findings indicate that the underlying mechanisms of cell degeneration and death are based on oxidative stress, mitochondrial dysfunction, inflammation, excitotoxicity, and apoptosis [2]. Intracellular proteinaceous inclusions such as Lewy bodies, which are composed mainly of alpha-synuclein, are common neural features of PD [3], [4]. Several genes have been found to cause monogenic forms of PD, and mutations in these genes result in neural dysfunction and neurodegeneration either by toxic gain-of-function or by loss of intrinsic protective functions [5].

The neurotoxin 1-methyl-4-phenyl-1,2,3,6-tetrahydropyridine (MPTP) causes permanent damage to DA neurons in the substantia nigra [6], [7]. MPTP-treated primates exhibit the neural damage and signs of PD such as moving tremors, immobility, muscle rigidity, and positional dysfunction that are observed in humans [8], [9], [10], [11]. Thus, MPTP is often used to establish PD model in various primate species, including baboons [12], macaques [6], [13], velvet monkeys [14], squirrel monkeys [7], [15] and common marmosets [16], [17], [18], for studying the pathophysiology, etiology, pathogenesis, and possible treatment options for PD. Preclinical (non-clinical) studies have shown that the common marmoset is a suitable model for not only the identification of effective pharmacological treatments but also the evaluation of neuroprotective therapies [19], [20], [21], [22], [23].

The behavioral characteristics of marmosets in the framework of preclinical PD studies have been described in previous studies [19]. In more detail, normal marmosets in individual living cages move freely, leap between mesh cage walls, and cling to the walls in the daytime, and are sedentary at night. As measured by infrared motion sensors, the daily locomotion count (spontaneous motor activity) for an individual normal marmoset in a cage remains stable for days. A stable but decreased level of locomotion count is observed consistently for several weeks or more following MPTP administration [19]; thereafter, the decreased level recovers to some degree. The stable level of decreased locomotion after MPTP administration is considered to be an objective and quantitative measure of immobility as a fundamental syndrome of PD [24]. In addition, this level provides a sensitive baseline for evaluating the beneficial effects of drugs in enhancing recovery [25]. Although the locomotion measurement in the MPTP-treated marmoset model is emphasized in the present study, observations of gross behavior are also necessary in order to obtain a general profile of the total behavioral manifestations including PD-like signs by MPTP or improvements by beneficial treatments and to avoid overlooking important aspects of behavioral changes [19]. Furthermore, observations of gross behavior can help to exclude irrelevant decreases (e.g., nonspecific depression, weakening, etc.) or increases (e.g., hyper-excitation by excessive dose of L-dopa, etc.) in locomotion counts owing to factors other than direct action of MPTP or recovery by beneficial treatments.

PET is an important technique for studying the changes in neural mechanisms underlying behavior [26], [27], [28]. PET imaging of the brains of MPTP-treated cynomolgus or rhesus monkeys showed that the binding of DA transporter (DAT) ligand and the utilization of L-3,4-dihydroxyphenylalanine (L-dopa) progressively decreased in the striatum upon MPTP administration and the manifestation of parkinsonism [29], [30], [31]. These studies used PET with markers of DAT or L-dopa utilization to identify the onset threshold of behavioral signs for the early diagnosis of presymptomatic PD. Various DAT ligands have been used to detect DA nerve degeneration in studies on MPTP-treated primate models of PD [30], [32]. Among them, the DAT-selective ligand [^11^C]PE2I has been used to investigate MPTP-treated cynomolgus monkeys [29], [33]. DATs are located on DA nerve terminals, and when MPP^+^, a conversion product of MPTP, causes the degeneration of DA nerve terminals in the nigrostriatal system, neither DAT nor any other site is available for binding [^11^C]PE2I. Thus, [^11^C]PE2I serves as a marker of DA nerve degeneration.

Studies on nigrostriatal nerve degeneration measured by PET in combination with behavioral observations in the MPTP-treated cynomolgus monkey have been already reported [30], [34]. Nevertheless, the present PET study using the MPTP-treated marmoset is significant because of its promising potential application in preclinical studies to evaluate not only acute drug therapies bur also neuroprotective drug therapies and brain neural implants. Furthermore, the recent development of transgenic marmosets [35] has enhanced the significance of PET studies in the MPTP model in this particular species, as the results can be a useful reference for evaluating the phenotypes of transgenic marmosets carrying genes of human alpha-synuclein.

In additional remarks on the common marmoset as an experimental primate, the body size of the marmoset is small comparable to that of the adult rat but its brain structure is highly developed [36], making this primate an excellent model for preclinical studies of various neuropsychiatric conditions. The marmoset is easy to handle owing to its even-tempered nature and has many attractive behavioral traits, including a keen interest in a variety of sensory stimuli. One important feature of the marmoset for breeding as an experimental primate is its fertility and productivity: a female marmoset sexually matures in 1.5 years and delivers two to four offspring twice a year to generate an average of 40 offspring in its 10-year lifetime as an experimental animal, compared with 36–48 offspring produced by a female rat over its usual 2-year lifetime in a laboratory situation. This aspect of marmoset primate biology makes it appealing for studies in laboratory animal science, genetic engineering, and regenerative medicine. Marmosets are currently being used as genetically modified primates models for diseases such as PD and Alzheimer’s disease [35], [37], [38], [39].

Taking into account the possible wide-ranging use of the common marmoset in preclinical studies, the MPTP-marmoset model needs to be characterized in terms of *in vivo* neurodegeneration and parkinsonism, especially because this model is serving as a reference control for evaluating transgenic PD marmosets. Therefore, the present study examined the relationship between decreased locomotion as an objective and quantitative measure of immobility and dopaminergic neurodegeneration in the striatum as revealed by PET scans.

## Materials and Methods

### Animals

Five male common marmosets (*Callithrix jacchus*), obtained from CLEA Japan, Inc. (Tokyo, Japan), were used in this study. These marmosets received subcutaneous (s.c.) administration of MPTP, and the behavioral consequences were observed at the Central Institute for Experimental Animals (CIEA) Then, the animals were sent to the National Institute of Radiological Sciences (NIRS) for PET scanning and analysis. Another five common marmosets, obtained by NIRS from the same source, were kept at NIRS as MPTP-free (control) marmosets during the entire experimental period.

The present study was conducted in strict accordance with the recommendations in the Guide for the Care and Use of Laboratory Animals, National Institutes of Health. The study protocol was reviewed and approved by the Institutional Animal Care and Use Committee of CIEA (CIEA approval no. 07028A). The criteria used by the committee complied with the Japanese Law for the Humane Treatment and Management of Animals. The maintenance and handling of marmosets at NIRS were also in accordance with institutional guidelines, and the Animal Ethics Committee of NIRS approved the PET study.

At CIEA, the marmosets were housed in individual stainless steel living cages (30×50×48 cm) with wire mesh floors, each enclosed by a sound attenuation box. The animals were supplied with a balanced diet (CMS-1M; CLEA Japan. Inc.) and *ad libitum* tap water from feed valves. The ages and body weights of the five marmosets at CIEA on the first day of MPTP administration were (mean ± standard deviation) 3.7±1.6 years and 340.4±20.3 grams, respectively. During MPTP administration and the subsequent 2-week period, supplements were given to compensate for anorexia and dehydration caused by acute MPTP toxicity. Blocks of CalorieMate (Otsuka Pharmaceutical Co., Ltd., Tokyo, Japan) were given in addition to normal daily food, and a mixed solution of Meibalance (7 mL; Meiji Dairies Corp., Tokyo, Japan) and Cakesia (Morinyu Sunworld Co., Ltd., Tokyo, Japan) was given orally by gavage. An electrolyte fluid, KN1A (Otsuka Pharmaceutical Co., Ltd., Tokyo, Japan), was also subcutaneously administered to all animals during MPTP administration and the subsequent 2-week period. The temperature and humidity in the animal room at CIEA, recorded each morning prior to cage washing, were 24–27°C and 53–76%, respectively. The room was illuminated from 9 a.m. to 9 p.m.

At NIRS, the five MPTP-treated marmosets sent from CIEA and the five MPTP-free marmosets kept at NIRS were housed in individual stainless steel cages with wire floors similar to those at CIEA and were maintained in a manner similar to that at CIEA. The ages and body weights of the five MPTP-treated marmosets on PET scan days were 4.7±1.2 years and 331.4±47.8 grams, respectively. The ages and body weights of the five MPTP-free marmosets on PET scan days were 4.0±2.0 years and 338.4±31.8 grams, respectively.

### MPTP Administration

MPTP hydrochloride powder (Sigma-Aldrich Co., St. Louis, MO, USA) was dissolved in physiological saline (Otsuka Pharmaceutical Factory, Inc., Tokyo, Japan) and was administered to each marmoset at a dose of 2 mg/kg in a volume of 1 mL/kg. The MPTP administration regimen (2 mg/kg s.c. per day for 3 consecutive days) was repeated two to four times in each marmoset at intervals of several months for 1.5 years, resulting in cumulative MPTP doses of 12–20 mg/kg. One MPTP-treated marmoset received only 2 days of MPTP administration during the 3^rd^ and 4^th^ regimens because the third dose of each regimen was canceled due to excessive toxicity (see cumulative MPTP dose in Table 1).

**Table 1:** Binding potentials (BP_ND_) of [^11^C]PE2I.

Group	Subject No.	BP_ND_ of Putamen	BP_ND_ of Caudate	Locomotion#1	Dose#2
MPTP-TREATED	CM22	0.94	0.82	0.25±0.05	18.0
	CM24	1.37	1.57	0.43±0.08	12.0
	CM25	1.07	1.37	0.28±0.05	18.0
	CM29	0.37	0.57	0.12±0.02	20.0
	CM33	0.61	0.85	0.15±0.03	18.0
	Mean±SD	0.87*±0.39	1.04*±0.42	0.25±0.12	17.2±3.03
MPTP-FREE	CM9	3.18	3.41	-	-
	CM10	3.54	3.04	-	-
	CM11	6.60	5.70	-	-
	CM16	6.11	5.22	-	-
	CM18	4.47	3.81	-	-
	Mean±SD	4.78±1.52	4.24±1.16	-	-

Binding potentials in the striatum (putamen and caudate) of brains of MPTP-treated and MPTP-free common marmosets are presented.

#1 :Ratios of mean daily locomotion counts of the post-MPTP period to those of pre-MPTP period (mean ± SD).

#2 :MPTP cumulative doses (mg/kg).

* :p<0.05 against MPTP-free marmosets (Bonferroni test). SD: Standard deviation.

Fatal DA neural degeneration at the striatum was reported by our autoradiography study [19] after the single MPTP administration regimen in the case of the common marmoset. The marmosets in the present PET study, however, received several MPTP regimens. The reason was that the marmosets in the present study were utilized from those used in other study after its completion. The purpose of the other study was to observe behavioral recovery after each MPTP administration regimen.

### Behavior Observations

Daily locomotion of each marmoset in its individual cage was continuously recorded by an infrared motion sensor (O’Hara and Co., Tokyo, Japan) attached to the cage ceiling. The sensor detected spatial shifts of thermal sources such as body parts of the marmosets. CIEA ACTSCAN software (Prime Lab, Inc., Tokyo, Japan) was used to record and analyze locomotion data [19]. In addition, the grossly observable general behaviors (gross behaviors) listed on the CIEA dysfunction score form, including moving tremor, hypoactivity, decreased vocalization, excessive blinking, lack of eye-tracking, lack of biting a pencil, and rough movements, were visually evaluated by experienced observers through a one-way mirror. Each of the 12 items listed on the form was recorded as observed (1) or not observed (0), and the dysfunction score was the total number of observed behaviors. Details have been described elsewhere [19].

### PET Scan and Analysis

PET scans of MPTP-treated marmoset brains were performed once for each marmoset at 9–13 months after the final MPTP administration, using [^11^C]-N-(3-iodoprop-2E-enyl)-2-carbomethoxy-3-(4-methylphenyl)nortropane ([^11^C]PE2I) as a DAT ligand. [^11^C]PE2I was synthesized by *O*-methylation from its precursor by reduction of [^11^C]CO_2_ with LiA1H_4_ in an inert atmosphere using specially designed equipment and was automatically purified at NIRS [29]. The radiochemical purity of the tracer ligand was >95%. The specific radioactivity at the time of administration was 55.1 to 185.9 GBq/µmol.

PET imaging of the marmoset brain using this ligand was performed with a microPET FOCUS 220 system (Siemens Medical Solutions USA, Knoxville, TN, USA). This system yields a 25.8 (transaxial)×7.6 cm^2^ (axial) field of view with a spatial resolution of 1.3 mm full width at half maximum at the center of the field of view [40]. During PET scanning, each marmoset was continuously anesthetized with 1.5–2.0% isoflurane (flow rate, 0.5 L/min; Abbott Japan Co., Ltd., Tokyo, Japan).

List-mode emission scans were performed for 90 min immediately after a single bolus administration of [^11^C]PE2I administration via the crural vein. The dose of [^11^C]PE2I administered ranged from 42.9 to 120.9 MBq. All list-mode data were sorted and Fourier rebinned into three-dimensional sinograms (frames × min: 5×1, 5×2, 5×3 and 12×5). Images were thereafter reconstructed using two-dimensional filtered back-projection with Hanning filter cut-off at the Nyquist frequency of 0.5 Hz.

The regions of interest (ROIs) were the caudate and putamen in the striatum, and the cerebellum was a reference region. These regions were individually identified using a magnetic resonance imaging (MRI) system. A 7 T MRI scanner (Magnet: Kobelco and JASTEC Japan; Console: Bruker BioSpin, Germany) with a volume coil for transmission (Bruker, Germany) and 2-channel phased array rat coil for reception (Rapid Biomedical, Germany) was used as a drawing counter of the brain for PET images and for determining ROIs. Marmosets were anesthetized with 1.5–2.0% isoflurane (Abbott Japan, Tokyo, Japan) administered through a facemask and immobilized with ketamine (30 mg/kg; Daiichi Sankyo Inc., Tokyo, Japan) during MRI. Two sets of transaxial multislice T2-weighted MR images (multislice spin echo; repetition time, 2500 ms; echo time, 40 ms; slice thickness, 1.0 mm; slice gap, 2.0 mm; matrix, 256×128; field of view, 38.4×38.4 mm^2^; number of excitations, 2; number of slices, 16) were acquired from a coronal section.

In the PET analysis, the radioactivity in each ROI was calculated for each frame and plotted versus time. Average values for ROIs in both the right and left hemispheres were used to increase the signal-to-noise ratio for the calculations. Regional binding potential relative to non-displaceable radioligand in the tissue (BP_ND_) was estimated by PMOD® (PMOD Technologies, Ltd., Zurich, Switzerland) with a simplified reference tissue model [41], [42], as described in the following equation:
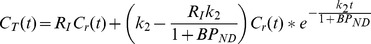
where C_r_(t) is the time course of radioactivity in the reference region (cerebellum), C_T_(t) is the time course of radioactivity in the target region, k_2_ is the efflux rate constant from the target tissue, R_I_ is the delivery rate in the target region compared with that in the reference region, and * is the convolution operator.

### Statistical Analysis

For each marmoset, the individual daily locomotion count after the MPTP regimens is expressed as a ratio to the mean daily count for the 7-day period just before the first MPTP regimen. Binding potentials (BP_ND_) of [^11^C]PE2I in the putamen and caudate were calculated for each marmoset in the MPTP-treated and the MPTP-free groups. Multiple analysis and the Bonferroni test were performed (*p*<0.05) using SPSS software (SPSS, Inc., Chicago, IL, USA). For MPTP-treated marmosets, the correlation coefficient between BP_ND_ in the striatum (putamen and caudate) and the mean ratio of daily locomotion counts was calculated with Pearson’s method using SPSS Statistics 19 software (IBM Co., Armonk, NY, USA). The correlation coefficient between BP_ND_ and cumulative MPTP dose was calculated in the same manner. Linear regression analysis was performed, and the coefficient of determination (R^2^) was calculated.

## Results

### Gross Behaviors and Locomotion

As grossly observable general behaviors, moving tremor, hypoactivity, and decreased vocalization were recorded in all five MPTP-treated marmosets for several weeks after each MPTP regimen. Excessive blinking was recorded in four MPTP-treated marmosets. Lack of stimulus tracking, lack of biting at a pencil, and rough movements were recorded in three MPTP-treated marmosets. Dysfunction scores (total number of observed items out of 12 items on the CIEA dysfunction score form; maximum possible score = 12) ranged from 5 to 7 in the five marmosets on the observation days.

The actual daily locomotion (count in mean ± SD) in all five marmosets for the 7-day period just before the first MPTP administration regimen was 13,290.6±4,039.9. Although the actual locomotion count varied among marmosets, the daily locomotion count was stable for each individual marmoset during the 7-day period. To minimize the count variability after MPTP administration, each daily locomotion count in an individual marmoset after the MPTP regimens is expressed as a ratio to the mean daily count for that marmoset during the 7-day period just prior to the first MPTP regimen, as described in the above statistical analysis section. The decreased ratio of daily locomotion counts was stable for several weeks or longer after each MPTP regimen, as reported previously [19]. In some marmosets, the decreased locomotion level recovered over time, but finally stabilized within several months or more after each MPTP regimen. The mean ratios of the locomotion counts for a 30-day period 3 months after the last MPTP regimen to the counts for the 7-day pre-MPTP period were 0.43 or lower (Table 1). The coefficient of variation (standard deviation/mean) across days during the 30-day period was ≤0.2, indicating that the locomotion counts (ratios to pre-MPTP period) across days during the 30-day period was stable for each individual marmoset.

### PET Analysis and Binding Potential

**Figure 1:**
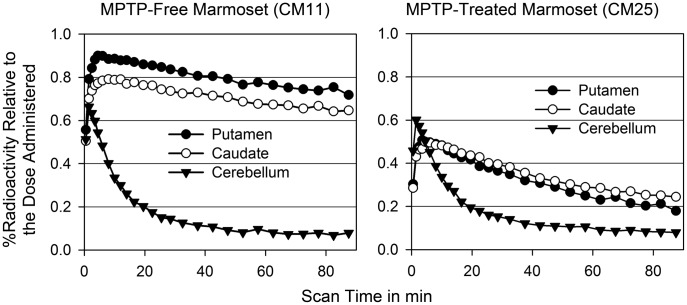
Radioactivity versus time curves in the brains of MPTP-free and MPTP-treated marmosets. A dopamine transporter ligand, [^11^C]PE2I, was intravenously administered to marmosets. The putamen and caudate in the striatum were the target regions, and the cerebellum was a reference region.

Representative time-radioactivity curves for [^11^C]PE2I from the PET analysis of MPTP-treated and MPTP-free marmosets are presented in Figure 1. The percentage of radioactivity in the cerebellum (reference ROI) relative to the [^11^C]PE2I dose markedly decreased in both MPTP-free and MPTP-treated marmoset brains. On the other hand, the radioactivity level in the striatum (caudate and putamen) of MPTP-free brains increased within 10 min after [^11^C]PE2I administration and then gradually decreased. In MPTP-treated brains, the level of radioactivity in the striatum did not increase as much as in the MPTP-free brains, and it declined rapidly with time. The curves of the other individual brains in each group were similar to the curve presented for the respective group.

**Figure 2:**
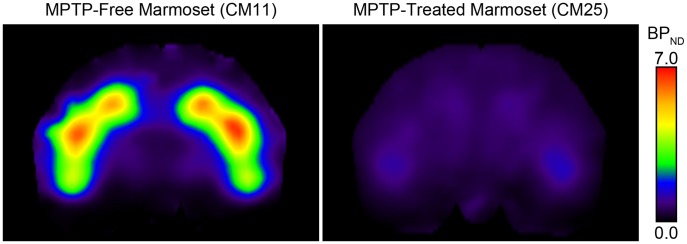
Representative parametric images. Coronal sections illustrating the binding potential (BP_ND_) of [^11^C]PE2I in the brains of MPTP-free and MPTP-treated marmosets are presented.

Figure 2 shows representative parametric images of BP_ND_ for [^11^C]PE2I in MPTP-free and MPTP-treated brains. BP_ND_ in the putamen and caudate appears to be high in the MPTP-free brains and very low in the MPTP-treated brains. The images of other individual brains in each group were similar to the image presented for the respective group. The BP_ND_ values for [^11^C]PE2I, expressed as the unit volume of voxels, in the caudate and putamen of individual MPTP-treated and MPTP-free brains are presented in Table 1. The mean BP_ND_ values in the caudate and putamen were significantly lower in MPTP-treated brains than in MPTP-free brains (Bonferroni test, *p*<0.05).

### Relationship between Binding Potential and Locomotion Counts

**Figure 3:**
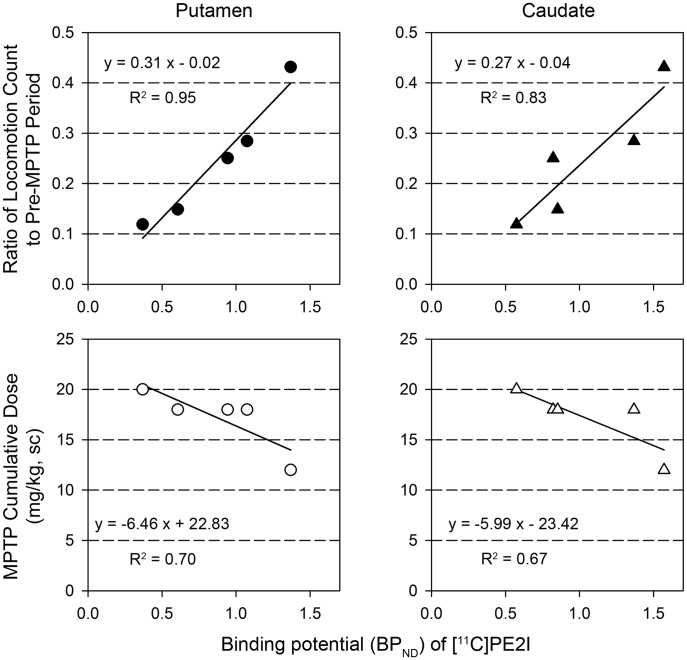
Regression lines between BP_ND,_ locomotion, and cumulative MPTP dose. The relationship between the binding potential (BP_ND_) of [^11^C]PE2I in the putamen or caudate and the daily locomotion count (relative to pre-MPTP counts) after cumulative MPTP administration was determined using the least squares method and is presented in the upper graphs. The relationship between BP_ND_ in the putamen or caudate and the cumulative MPTP dose is presented in the lower graphs.

To examine the relationship between striatal DA neuron degeneration and immobility as a central parkinsonian sign, the correlation coefficient between the BP_ND_ of [^11^C]PE2I in the striatum and the daily locomotion count (ratio to pre-MPTP period) was calculated in MPTP-treated marmosets (Figure 3). The daily locomotion count ratio and BP_ND_ showed a significant positive correlation in both the putamen (r = 0.98, *p*<0.01) and caudate (r = 0.91, *p*<0.05). The cumulative MPTP dose and ratio of daily locomotion count were negatively correlated (r = −0.91, *p*<0.05). The BP_ND_ and cumulative MPTP dose were not significantly correlated (−0.84 in the putamen; −0.82 in the caudate). The subjectively evaluated dysfunction score was not included in the correlation analysis because the score change was limited and because the score was not measured using a ratio scale (*i.e.*, the weight of each item was not adjusted) [43].

## Discussion

### Present Findings and their Implications

In the present study, MPTP-induced degeneration of DA neurons in the striatum of the small marmoset brain was clearly detected by noninvasive PET as reported by other studies using larger brains of macaque monkeys. The present study indicated that the degree of the striatal neural degeneration caused by MPTP was highly correlated to the decreased level of locomotion counts in the individual living cage as an objectively measurable index of immobility, one of the fundamental signs of parkinsonism. This finding provides a basic knowledge for preclinical evaluations of drugs and stem cell or neural implantation in this MPTP-treated marmoset model. Furthermore, the characterization of neurodegeneration and behavioral changes detected in the MPTP treated marmoset PD model can be a useful reference for assessing the phenotype of the transgenic marmoset carrying human PD gene being developed at present. When the transgenic marmoset is to be established as PD model with clear phenotype, it will have a great practical significance for understanding PD pathophysiology and for preclinical evaluations of various therapies for PD.

### MPTP-induced Marmoset Model Versus Macaque Model

MPTP-treated primate models of PD are useful for elucidating mechanisms of the disease, and for evaluating drug treatments and neural implants [44], [45]. The common marmoset is a convenient model primate because it exhibits fatal degeneration of nigrostriatal dopaminergic neurons after only a single MPTP regimen as reported by our autoradiograpy study [19]. In this previous study, bindings of [^11^C]PE2I, the same DAT ligand as used in the present PET study, at the caudate, putamen and substantia nigra of the marmosets were remarkably decreased after only single MPTP regimen (MPTP at 2 mg/kg, s.c., per day for 3 consecutive days) with marked PD signs such as immobility, moving tremor, muscle rigidity, positional dysfunction, etc. In the macaque model, repeated administrations of low dose MPTP with separated week intervals are necessary in several months or more for gradual degeneration of the dopaminergic neurons with the behavioral baseline of PD signs.

Another advantage of the marmoset as a PD model is the ease of measuring changes in locomotion of individuals in cages, as marmosets have stable high levels of locomotion before MPTP treatment and stable low levels after MPTP treatment. The sensitive recovery of the decreased level of locomotion of the MPTP-treated marmoset to a normal level is especially useful for detecting symptomatic improvement by drug administration. In our previous study (Ando, K., published in Japanese, 2004), MPTP-treated cynomolgus monkeys showed decreased level of locomotion counts in their individual living cages, but did not recover from the decreased level after intra-gastric administration of L-dopa at 2.5–20 mg/kg, although L-dopa at 5 and 10 mg/kg resulted in improved scores for visually observed gross behaviors. Therefore, it is stated that the improvement caused by L-dopa in the macaque was detected in the scores but not in the locomotion counts in terms of the attenuation of the MPTP-induced decreased level. The reason for the difference of the behavioral effects on locomotion by L-dopa between the marmoset and the macaque may be related to the fact that the marmoset is more moving in nature than the macaque in their individual cages with limited space.

Previous PET studies in MPTP-treated macaque monkeys have already demonstrated degeneration of nigrostriatal DA neurons and the consequent behavioral manifestations similar to the clinical hallmarks of human PD [20], [46], [47], [48], with a high correlation between nigrostriatal DA neurodegeneration and decreased motor activity [30], [34], [49], [50]. Among them, the present study may be the first in the marmoset with small brain to report a high correlation between DA neurodegeneration by PET and the behavioral signs of immobility. The present findings may give useful information to other investigators performing studies using PD model marmoset. As an example, transgenic marmosets carrying alfa-synuclein genes are developing at the moment. The manifestations of PD-like phenotypes of these transgenic marmosets are examined by noninvasive PET measurement and behavioral observations. In such situation, the present MPTP-treated PD marmoset model is playing a very important role as a reference control for evaluating phenotype of the transgenic PD marmosets.

### Baseline Level of Locomotion in MPTP-treated Marmosets

Although low levels of locomotion were maintained in individual marmosets for several weeks or more after MPTP administration regimen, the locomotion levels in some marmosets recovered over time, although not up to the pre-MPTP levels. The locomotion data used for the present analysis were obtained during a 30-day period 3 months after the last MPTP regimen. As evidenced by the low coefficients of variation (Table 1), the locomotion level during this period was stable for each marmoset and was free from acute toxic effects of MPTP. In another study that we performed using eight common marmosets (Ando et al, unpublished data), several PD signs and low locomotion counts were observed in MPTP-treated marmosets for longer than 1.5 years after the last MPTP regimen. Individual marmosets in these studies by us showed stable low levels of locomotion for above period. These studies may provide a rationale for using daily locomotion counts during a limited time period for the correlation analysis with BP_ND_ of PET analysis in the present study.

### Some Issue of the Present PET Measurement in MPTP-treated Marmosets

Noninvasive PET measurement is an effective technology for investigating progressive neural changes in the striatum in conjunction with parkinsonism behaviors [29], [31], [48]. In the present PET measurement, scan was performed only once in the marmosets, approximately 1 year after the last MPTP regimen. As no PET scans were performed before the MPTP regimens, the BP_ND_ values in the post-MPTP period were not compared to the pre-MPTP values for the standardization in order to minimize the bias of individual differences. However, the present statement about the results of PET measurement is based on the remarkably lower levels of BP_ND_ values of MPTP-treated marmosets than those of MPTP-free marmosets. The PET measurement was performed in an appropriate manner in NIRS with enough accumulated background of PET studies with high reproducibility and reliability using the present ligand in primates.

One more thing to be mentioned is that successive PET measurements were not performed during several MPTP regimens in the present marmoset study while successive measurements were usually performed in macaque PET studies. The marmosets in the present PET measurement were utilized from other behavioral study after its completion as described in the method section. Furthermore, in our autoradiography study described above, bindings of [^11^C]PE2I at the nigorstriatal regions of the MPTP induced PD model marmosets were remarkably decreased after only single MPTP regimen. Therefore, successive PET measurements during MPTP regimens in the marmoset may not have been necessary.

Despite the methodological issue, the present study demonstrated the significance of measuring long-term daily locomotion of individual marmosets in living cages for monitoring parkinsonian signs and the significance of noninvasive PET analysis for determining striatal neurodegeneration in this small primate. The present study provides a technical basis for future studies in the common marmoset using PET in combination with locomotion counts as an objective, quantitative, and sensitive measure of immobility, a key syndrome of PD.

### Conclusions

The degeneration of DA neurons in the striatum of the MPTP-treated marmoset was clearly detected by noninvasive PET scan and was highly correlated with decreased behavioral mobility. These results suggest that noninvasive PET measurements of DA neurons in conjunction with locomotion observations in marmosets may be useful in the preclinical evaluation of treatments for PD. Furthermore, the present study may be important in evaluating the validity of behavioral and neural phenotypes of transgenic marmosets following the introduction of human PD genes. MPTP-treated marmoset PD model can play a role of a reference control for transgenic PD model in its development process.
